# Sparing Sphincters and Laparoscopic Resection Improve Survival by Optimizing the Circumferential Resection Margin in Rectal Cancer Patients

**DOI:** 10.1097/MD.0000000000002669

**Published:** 2016-02-08

**Authors:** Metin Keskin, Adem Bayraktar, Emre Sivirikoz, Gülcin Yegen, Bora Karip, Esra Saglam, Mehmet Türker Bulut, Emre Balik

**Affiliations:** From the Istanbul University, Istanbul Faculty of Medicine, General Surgery Department, Millet Caddesi, Capa, Istanbul, Turkey (MK, AB, ES, MTB); Istanbul University, Istanbul Faculty of Medicine, Pathology Department, Millet Caddesi, Capa, Istanbul, Turkey (GY); Fatih Sultan Mehmet Education and Research Hospital, Department of General Surgery, İçerenköy—Ataşehir, Istanbul, Turkey (BK); Istanbul University, Oncology Institute, Millet Caddesi, Capa, Istanbul, Turkey (ES); and Koc University, School of Medicine, General Surgery Department, Rumelifeneri Yolu, Sarıyer, Istanbul, Turkey (EB).

## Abstract

The goal of rectal cancer treatment is to minimize the local recurrence rate and extend the disease-free survival period and survival. For this aim, obtainment of negative circumferential radial margin (CRM) plays an important role. This study evaluated predictive factors for positive CRM status and its effect on patient survival in mid- and distal rectal tumors.

Patients who underwent curative resection for rectal cancer were included. The main factors were demographic data, tumor location, surgical technique, neoadjuvant therapy, tumor diameter, tumor depth, lymph node metastasis, mesorectal integrity, CRM, the rate of local recurrence, distant metastasis, and overall and disease-free survival. Statistical analyses were performed by using the Chi-squared test, Fisher exact test, Student *t* test, Mann–Whitney *U* test and the Mantel–Cox log-rank sum test.

A total of 420 patients were included, 232 (55%) of whom were male. We observed no significant differences in patient characteristics or surgical treatment between the patients who had positive CRM and who had negative CRM, but a higher positive CRM rate was observed in patients undergone abdominoperineal resection (APR) (*P* < 0.001). Advanced T-stage (*P* < 0.001), lymph node invasion (*P* = 0.001) and incomplete mesorectum (*P* = 0.007) were encountered significantly more often in patients with positive CRM status. Logistic regression analysis revealed that APR (*P* < 0.001) and open resection (*P* = 0.046) were independent predictors of positive CRM status. Moreover, positive CRM was associated with decreased 5-year overall and disease-free survival (*P* = 0.002 and *P* = 0.004, respectively).

This large single-institution series demonstrated that APR and open resection were independent predictive factors for positive CRM status in rectal cancer. Positive CRM independently decreased the 5-year overall and disease-free survival rates.

## INTRODUCTION

Surgery remains the main stay of curative treatment for rectal cancer. The purpose of surgical treatment is to minimize the local recurrence rate, extend the disease-free survival period, and preserve patient quality of life. The total mesorectal excision (TME) technique described by Heald, which consists of resection of the rectum within the mesorectal envelope, allows for the removal of the mesorectum en bloc along with the fascia recti propria.^1,2^ The local recurrence rate in rectal cancer patients exceeded 25% before implementation of the TME technique, whereas the local recurrence rate was reduced to 4% to 5% with the implementation of TME.^2–4^ Neoadjuvant radiotherapy, which was pioneered by the Swedish Study Group, is more efficient with respect to postoperative sequelae, as surgery results in trauma to the tissue, which results in poor perfusion and oxygenation. Neoadjuvant radiotherapy improves local recurrence and survival rates by reducing the tumor size and stage.^4–8^ Subsequent studies demonstrated that chemotherapy treatment with neoadjuvant radiotherapy resulted in better outcomes regarding local recurrence and survival rates when compared with radiotherapy alone.^6–9^ Contemporary neoadjuvant treatment of middle and distal rectal tumors in a neoadjuvant setting (stages II–III) is the gold standard to increase the effectiveness of radiotherapy, provide negative surgical margins, enhance the chance of sphincter-preserving surgery and improve local recurrence and survival rates.

Quirke et al^10^ defined the concept of the circumferential radial margin (CRM) as the distance from the tumor to the mesorectal fascia, which challenged the belief that local recurrences originate from the distal margin of the anastomosis site. This group demonstrated that CRM is a prognostic factor for local recurrence and the majority of local recurrences originate from the residual tumor remaining on the pelvic wall after the initial resection (ie, CRM involvement).^10^ Reduction of local recurrence rates can be achieved by increasing surgical experience and skills as well as by increasing our understanding of the CRM concept and the significance of the removal of the mesorectum en bloc through anatomical and pathological assessments. TME and the status of the CRM are the key parameters for a successful resection. The former parameter provides an evaluation of surgical quality, and the latter parameter predicts local recurrence, systemic spread, and survival rates.^5,11,12^

In this study, we investigated the effects of a positive CRM on local recurrence and survival rates based on mesorectal excision completeness as well as the causes of CRM positive or negative status.

## MATERIALS AND METHODS

**FIGURE 1:**
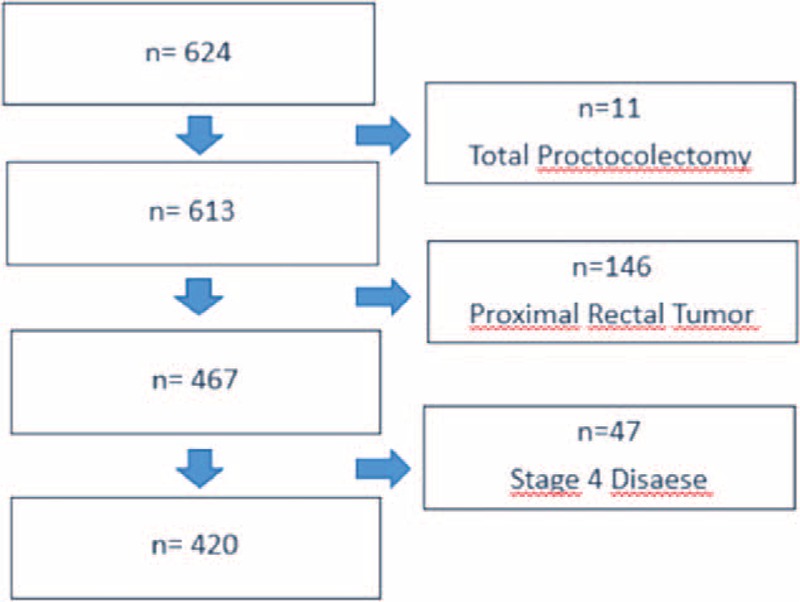
Exclusion criteria.

Approval was obtained from the ethics committee (No: 2012/741-1059), and patients with histologically confirmed tumors located in the middle and lower rectum, who had undergone curative surgery between January 2005 and December 2012, were included in the study. Stage IV cases at the initial diagnosis and patients with synchronous colorectal tumors or tumors on the proximal rectum were excluded (Figure 1). The data were recorded in Microsoft Excel® software and were evaluated retrospectively using SPSS® software (Statistical Package for Social Sciences, Inc., Chicago, IL, ABD) for Windows, version 21.0.

Gender, age, body mass index (BMI), tumor location (middle or distal), neoadjuvant treatment, surgical technique, surgery type, tumor diameter (mm), tumor invasion depth (T), circumferential margin, lymph node invasion (N), and completeness of the mesorectal resection were obtained from the database. Evaluations of the completeness of mesorectum excision were standardized in our Pathology Department after 2007. Therefore, the series included in the analysis was restricted to 371 patients who satisfied the criteria for completeness of mesorectum resection. CRM was recorded as positive in cases with a ≤1 mm distance between the tumor and the fascia propria recti.^13^

Informed consent was obtained from all patients. The preoperative staging evaluation included abdomen and chest computerized tomography scans, *pelvic magnetic resonance imaging, with or without endorectal ultrasonography*. Neoadjuvant chemoradiotherapy or radiotherapy was performed in patients with T3> tumors and/or N (+) on imaging. Surgery was performed 8 weeks after neoadjuvant chemoradiotherapy and 4 weeks after short-term radiotherapy.

All patients underwent TME surgery with curative intent by 6 experienced colorectal and laparoscopic surgeons who completed their learning curve in laparoscopic surgery between 2002 and 2005. Laparoscopic surgery was recommended to all patients. Some patients underwent open surgery because they did not consent to laparoscopic surgery or had previous abdominal surgery. During the surgery, inferior mesenteric artery and vein were ligated at their origins. Then, rectum was mobilized throughout the Holly Plan to the levator muscle level, adhering to the principles of TME. In patients, who underwent sphincter preserving surgery, anastomosis was performed by circular stapler or it was done by hand sewn. The techniques in the intra-abdominal section were similar for abdominoperineal resection (APR) procedure. After mobilizing the rectum up to the levator muscle, end colostomy was created and abdomen part of the operation was finished. The wide perineal incision covering sphincters was done. Perineal dissection was performed up to tip of coccyx posteriorly, and then it was completed anteriorly and laterally. Specimens were examined in 0.5-cm tissue sections after at least 72 hours of fixation to ensure proper circumferential margin examination. Adjuvant treatment, consisting of 4 doses of 5-fluorouracil/folic acid, was given to all patients who received neoadjuvant treatment as well as to patients with pT3 and patients with lymph node invasion. Clinical follow-ups were obtained upon clinical visits, rehospitalization and the other procedures such as endoscopy, radiological studies. The missing data of the patients about possible relapse, metastasis, and survival rates; whom were not found on the records, were collected and updated according to the conversation with the patients through the phone calls and clinical visits.

### Statistical Analysis

The patient cohorts with negative and positive CRM associated with mid- and distal localized rectal tumors were compared for differences in demographic, clinical, and pathological characteristics using bivariate analysis. The Chi-squared test or Fisher exact test was used to compare categorical variables. Continuous variables were examined for normality of distribution using the Shapiro–Wilk test. Student *t* test was used to analyze normally distributed variables, and the nonparametric Mann–Whitney *U* test was used for the analysis of nonnormally distributed values.

All variables in the bivariate analyses were entered into a forward logistic regression model to correct for selection bias and to identify independent predictors of CRM. Overall and disease-free 5-year survival rates for patients who completed 60 months of follow-up after recovery from surgery were analyzed in our comparison of patients with negative- and positive-CRM using the Mantel–Cox log-rank sum test. Patients with local recurrence and distant metastases were compared for overall and at 5-year follow-ups using bivariate analysis.

## RESULTS

Analysis of the patient demographic characteristics revealed that 232 patients were male (55%), and 188 patients were female (45%). The mean age of the patients was 58 years (range 19–91 years). A total of 347 (83%) patients received neoadjuvant treatment. Laparoscopic surgery was performed on 327 (78%) patients. The rate of sphincter-preserving surgery was 65% (n = 272). A total of 31 cases (7.4%) exhibited positive CRMs as verified by pathological examination. Comparisons between patients with negative and positive CRMs revealed no significant differences in demographics, patient characteristics, tumor location (mid or distal), neoadjuvant therapy, or laparoscopic resection; however, a higher positive CRM rate was observed in patients who received APR (14.9 vs. 3.3, *P* < 0.001) (Table 1). APR rate was found similar between patients operated with laparoscopic surgery and patients operated with open surgery [laparoscopic: 35.2% (115/327) and open: 35.5% (33/93); *P* = 1.000].

**TABLE 1:**
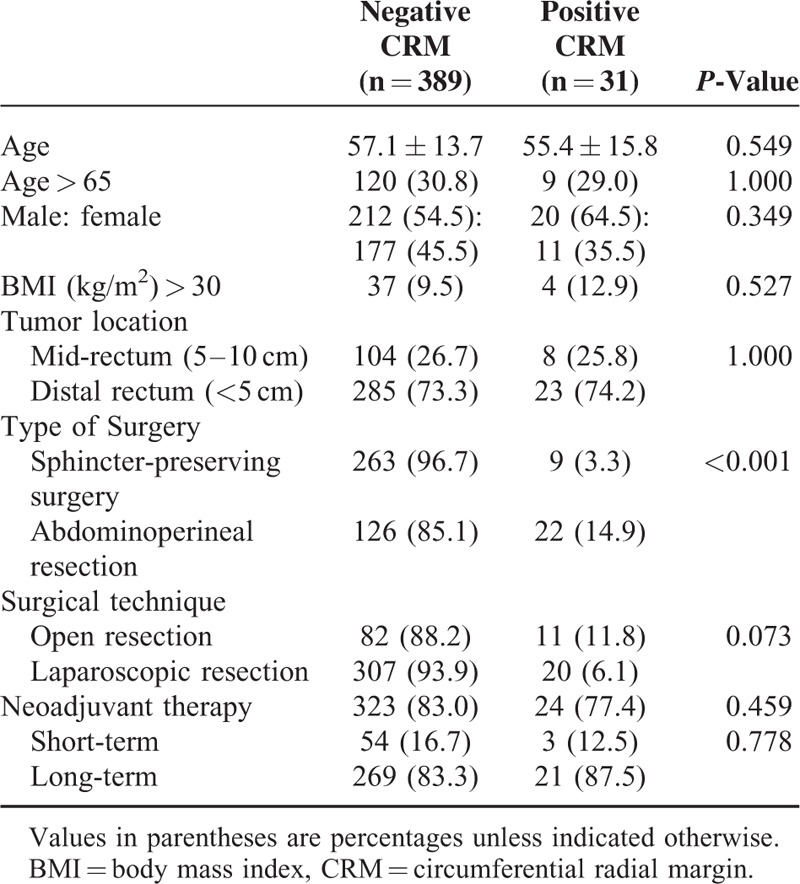
Demographics and Patient Characteristics

Comparisons of pathological risk factors revealed that advanced T-stages (*P* < 0.001), lymph node invasion (*P* = 0.001) and incomplete TME rates (*P* = 0.007) were encountered significantly more often in patients with positive CRMs (Table 2). Except lymphatic invasion, minor pathologic features of tumors (perineural invasion, vascular invasion, etc.) did not effect positive CRM status, but data of these factors was obtained only 37.5% of patients. Since patients were not randomized for surgical technique (open or laparoscopic) in the present study, we evaluated tumor's diameter relative to surgical technique (open or laparoscopic) to show that surgeons did not use tumor diameters as a criteria for deciding about the surgical technique. The tumor diameter (tumor size ≥5 cm) did not differ between laparoscopic and open surgery group (laparoscopic: 33.8% vs. open: 23.6; *P* = 0.082).

**TABLE 2:**
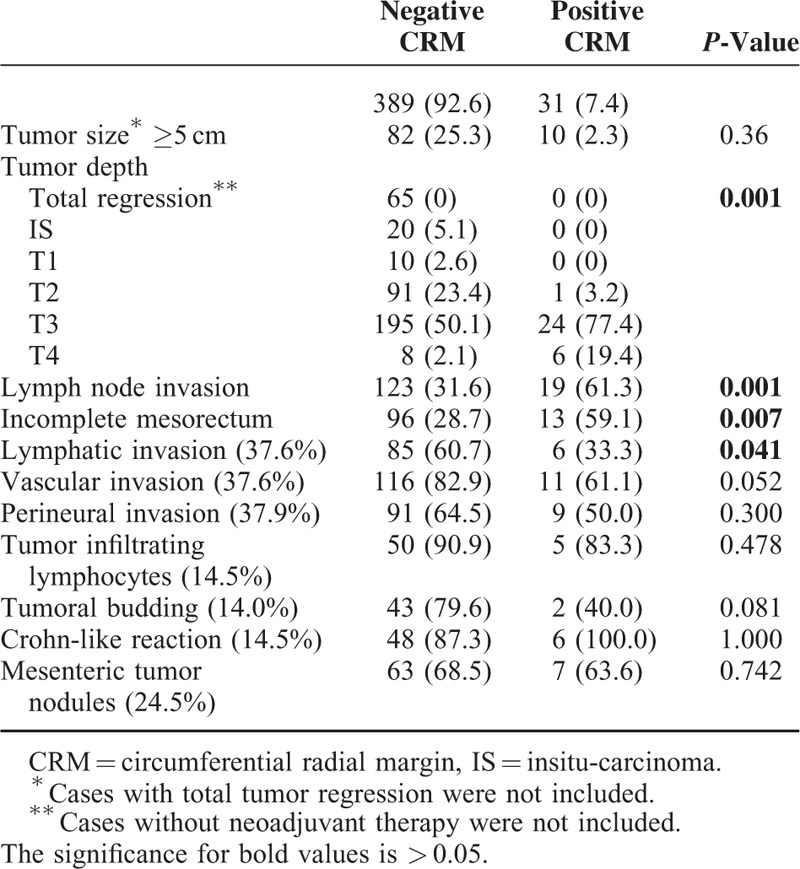
Pathologic Risk Factors

All variables in the bivariate analyses were entered into a forward logistic regression model, which revealed that APR and open resection were independent predictors of positive CRM (Table 3).

**TABLE 3:**
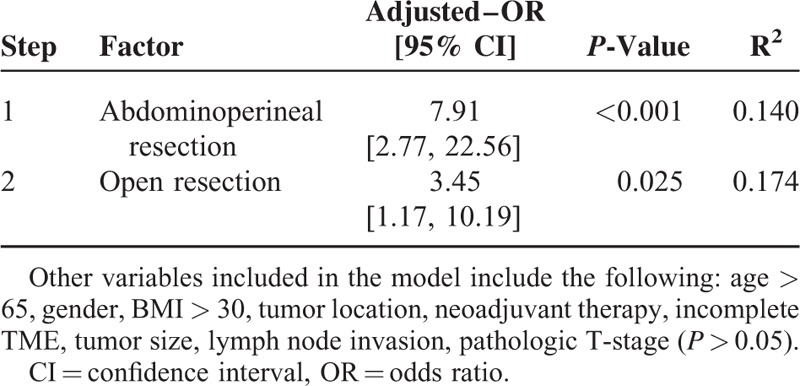
Independent Predictors of CRM Involvement

The follow-up rate for this study was greater than 95%. The mean follow-up period was 51.4 ± 24.4 months. Twenty-seven (6.4%) patients experienced local recurrence, and 72 (17.1%) patients experienced metastases to distant organs.

Overall, 5 (16.1%) patients with positive CRMs and 22 (5.7%) patients with negative CRMs experienced local recurrence (*P* = 0.040). Overall, 12 (38.7%) patients with positive CRMs and 60 (15.4%) patients with negative CRMs experienced metastases to distant organs (*P* = 0.002).

**FIGURE 2:**
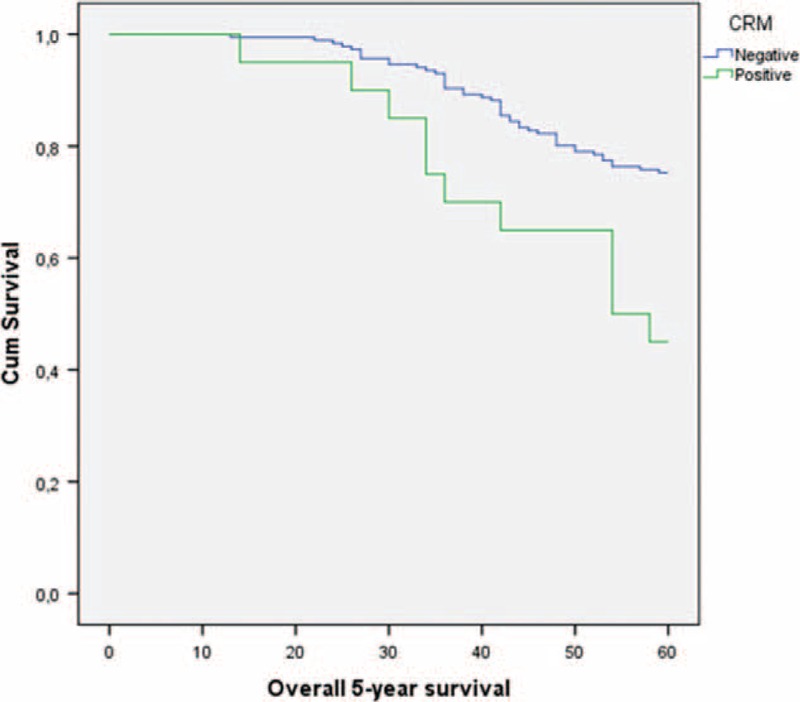
Five-year overall survival curves according to CRM using Kaplan–Meier analysis.

**FIGURE 3:**
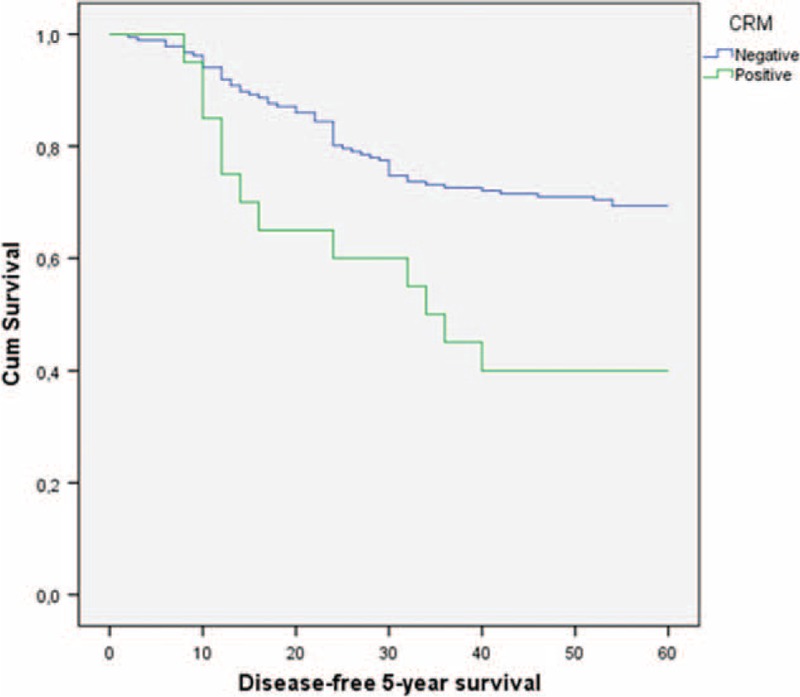
Five-year disease-free survival curves according to CRM using Kaplan–Meier analysis.

Overall and disease-free 5-year survival rates were analyzed based on negative and positive CRM status. Positive CRM was associated with significantly decreased 5-year overall and disease-free survival rates (Table 4) (Figures 2 and 3).

**TABLE 4:**
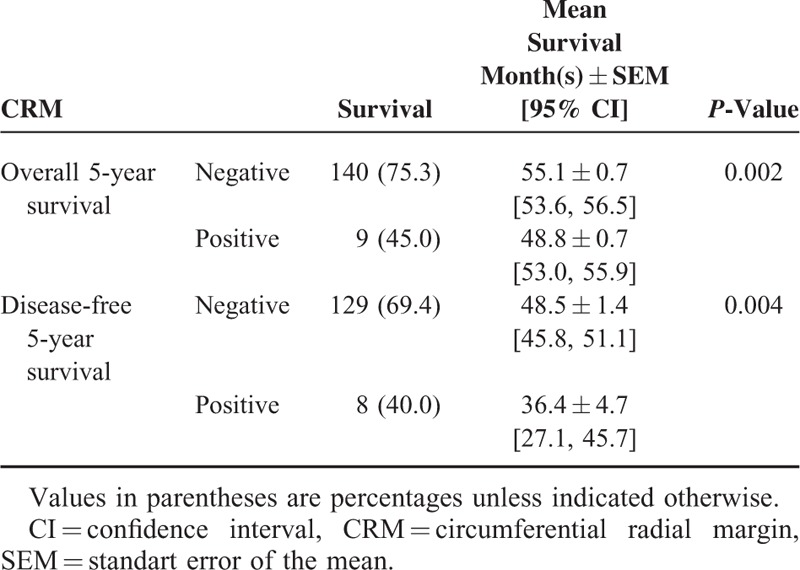
Survival Analysis (Log-Rank Sum Test)

Cox-regression analysis was performed to identify independent predictors for survival based on surgical technique. When laparoscopic resection, incomplete mesorectum, APR and CRM status were entered into a stepwise logistic regression model, CRM involvement was the only independent predictor of survival (overall 5-year survival AOR: 3.6 [1.6, 8.2], *P* = 0.002; disease-free 5-year survival AOR: 3.1 [1.4, 7.0], *P* = 0.006).

## DISCUSSION

The factors influencing rectal cancer outcomes are classified as tumor, patient, and surgeon related. Surgical skills and experience, comprehension of the significance of 2 essential concepts, and the selection of the appropriate surgical technique are accordingly important for the reduction in the rate of local recurrence. These concepts include the removal of the mesorectum en bloc (TME) and the achievement of negative circumferential margins. A significant correlation has been observed between TME performance and the subsequent development of local recurrence.^2,14^ The notion of TME has been a major concept associated with the reduction of local recurrence, but differences in the competence of the surgeon performing the technique should not be overlooked.^15^ CRM status initially proposed by Quirke et al^10^ is the most influential factor for local recurrence rates. Following TME, in last 15 years, we have observed two major variations in rectal cancer treatment, utilizing neoadjuvant treatment and performing minimal invasive surgery. Although many previous studies indicated CRM positivity as a prognostic factor on rectal cancer long-term outcomes, recent study from Sweden claimed that the relationship between CRM (+) and local recurrence is less important than previously stated and they contributed it to the innovation of pre- and postoperative oncological treatment.^10,13,14,16^

The present cohort study, in which multiple factors including these new treatment approaches with demographic, clinic, operative, and pathologic data were evaluated to determine the predictive factors on CRM positive in extraperitoneal rectal cancer, stated that tumor invasion depth, lymph node positivity, incompleteness of mesorectal integrity and APR are associated with positive CRM whereas demographic features, surgical technique, tumor location, and neoadjuvant therapy method have no effect on CRM positivity. The logistic regression analysis revealed that APR increases CRM positivity by 7.9 times as open surgery increases it by 3.45 times. Cox-regression analysis showed that CRM was the only independent predictor on survival.

The correlation between positive CRM status and age, gender, or BMI has been previously investigated, but, as in our study, a statistically significant correlation was not observed.^17–19^ Rullie et al^20^ found that the median length of CRM can be extended with neoadjuvant treatment. In contrast, many studies have demonstrated that neoadjuvant treatments did not significantly affect CRM positivity.^17,21,22^ We also did not identify a positive effect of neoadjuvant treatment on CRM positivity. The sort of short- or long-term neoadjuvant treatment also did not impact on CRM positivity. Although some studies identified that tumors with diameters ≥5 or ≥4 cm were associated with increased positive CRM status, controversial study showed no significant correlation between tumor diameter and CRM positivity.^18,19,23^ We did not found any correlation between tumor diameter and CRM positivity. We also added tumor's diameter into the multivariate analyses and we still did not identify a significant correlation between tumor diameter and CRM positivity.

It has been shown that increasing T stage and lymph node invasion (N+) were associated with CRM positivity.^17,19,21,24^ Our study also demonstrated that significantly higher rates of CRM positivity were seen in patients with T3 and T4 tumors or N+ tumors. However, multivariate analysis revealed no significant correlation between these pathological features and CRM positivity. We believe that the reason for these results can be explained with utilizing neoadjuvant treatments for patients who had local advanced rectal cancer. Many studies demonstrated that surgery associated with the complete removal of the mesorectum reduces positive CRM status and local recurrence rates.^21,25–27^ An MRC 07 trial reported a local recurrence rate of 1% in cases of stage III rectal tumors in which complete removal of mesorectum was achieved.^28^ A statistically significant correlation between incomplete TME and positive CRM status was also observed in the present study.

The primary concerns associated with the laparoscopic surgical treatment of rectal cancers are distal surgical margin and CRM. Proper laparoscopic colorectal surgery requires an adequate level of experience in colorectal and laparoscopic surgeries. The learning curve for these techniques is long and difficult, which slows down the widespread use of laparoscopic rectum cancer surgery, increases the uncertainty of the oncological outcomes and unearths the need for high-capacity experienced centers to competently apply the technique. In the COLOR II and MRC CLASICC trials, no significant differences were found in CRM positivity between conventional and laparoscopic-assisted surgery of rectal tumors but COLOR II study stated that CRM positivity was significantly higher in the open surgery group than the laparoscopic surgery group for distally located tumors.^29,30^ In our previous study, which included proximal, middle, and distal tumors, CRM positivity did not differ between laparoscopy and open surgery groups.^31^ However, the mesorectum becomes thinner in the distal rectum and there is no mesorectum to act as a barrier at the anorectal area and many studies suggested that positive CRM involvement decreased as the distance from the anal verge increased.^21,32^ Therefore, in the present study, patients with proximal located rectal tumors were excluded and only patients with extraperitoneal located rectal tumors were included. Whereas surgical technique (open or laparoscopic) did not effect on positive CRM status, no significant correlation was found between tumor location (middle or distal) and positive CRM status. However, the logistic regression model revealed that open surgery was the second independent risk factor for positive CRM status with APR. Multivariate analysis also indicated that open surgery increased CRM positivity 3.45-fold (*P* < 0.001). A randomized trial COLOR II showed the same result as the present study relative to CRM positivity. They found that laparoscopic surgery was associated with lower CRM positivity in patients with distal located rectal tumors and they also observed less local recurrence in laparoscopic group than open surgery group.^33^ We believe that this result was due to the favorable effect of laparoscopic surgery, which can provide the ability to conduct precise dissection under an enlarged endoscopic view like the same comment was stated in COLOR II trial.^33^

Although the outcomes of rectal cancer treatment have improved remarkably with new treatment strategies in last 20 years, the worse local recurrence rate has been still observed in patients treated with APR compared to patients treated with sphincter-preserving surgery secondary to high CRM positivity as reported previously.^17,18,21,34^ CRM positivity in the present study was significantly higher in patients who underwent APR. In the multivariate analysis, APR was found an independent risk factor for positive CRM involvement and increased CRM positivity 7.9-fold. Subgroup analyses in the present study revealed that incomplete mesorectum excision, which is an another negative factor for CRM positivity, resulted in a 2-fold increase in the APR group (*P* < 0.001). The achievement of negative surgical margins is more difficult as the tumor approaches the anal verge because of the lack of mesorectal tissue and the restriction of the pelvic outlet on the sidewalls by pelvic bones.^32,34,35^ To handle this issue some surgeons recommended extralevator APR, a wider resection including the levator muscles,^36,37^ but the benefit of extralevator APR is not clear in the literature. Recently, the retrospective study from Denmark and the systematic review from China revealed that extralevator APR did not improve CRM positivity compared to standard APR.^35,38^ In the present study, extralevator APR technique was not used on patients.

Positive CRM status is an influential prognostic factor for local recurrence, systemic spread, and survival rates.^10,13,24^ Wibe et al^14^ observed a local recurrence rate of 22% in patients with positive CRMs and 5% in patients with negative CRMs in their study. Quirke et al^21^ showed that CRM positivity negatively affected local recurrence and 3-year disease-free survival rates. However, the multivariate analysis, in which all parameters were included, revealed that N stage, T stage, completeness of mesorectal excision and tumor location were risk factors for local recurrence but positive CRM was not a risk factor.^21^ This result has been debated in the literature because neoadjuvant treatment can compensate for poor surgical skills and CRM positivity.^26^ Overall, local recurrence rates and distant metastasis in the present series were statistically higher in patients with positive CRM status.

Our previous study found that positive CRM was significantly associated with overall and disease-free survival rates.^31^ Kennelly et al^24^ demonstrated that CRM positivity decreased survival. CRM positivity was a significant factor on survival rates and was even more important than the TNM staging system for prognosis when combined with lymph node invasion status.^26,39^ In the present study, it was found that positive CRM status significantly reduced overall and disease free 5-year survival rates.

The main limitation of the present study is the retrospective nature of our analysis. The goal of curative rectal cancer surgery is to achieve negative CRMs and to protect sphincter function. This goal encumbers the design of prospective randomized trials that evaluate predisposing factors for positive CRM in rectal cancer surgery. Another limitation to our study is the evaluation of the completeness of mesorectum integrity, as this factor was only evaluated at our institute after 2007. We would like to emphasize that the present study, in which 78% of the patients underwent laparoscopic surgery, presents results from a single high-volume center in colorectal surgery. We think that this is important because it has been previously shown that the hospital volume influences CRM positivity rate and long-term outcomes in rectal cancer treatment.^40^

## CONCLUSIONS

Our retrospective analyses, in mid- and distal rectal cancer patients, revealed that tumor invasion depth, lymph node positivity, incomplete mesorectal excision, and APR increased positive CRM status. In the logistic regression analysis of our large single-institution series, APR and open resection were independent predictors of CRM status for extraperitoneal rectal cancer. Significant increases in distant organ metastasis and local recurrence were observed during the follow-up period in the CRM-positive group of patients. Overall and disease-free 5-year survival rates were significantly decreased in patients with positive CRM status. Evaluation of the impact of the surgical techniques on survival rates revealed that positive CRM was the only independent predictor of survival.
